# CT and MRI features of hepatic epithelioid haemangioendothelioma: a multi-institutional retrospective analysis of 15 cases and a literature review

**DOI:** 10.1186/s13244-022-01344-y

**Published:** 2023-01-05

**Authors:** Lianmei Luo, Zeyu Cai, Sihui Zeng, Lizhu Wang, Zhuang Kang, Ning Yang, Yaqin Zhang

**Affiliations:** 1 Department of Radiology, The Fifth Affiliated Hospital, Sun Yat-Sen University, No. 52 Meihua Dong Road, Zhuhai, 519000 Guangdong China; 2 Department of Radiology, State Key Laboratory of Oncology in South China, Collaborative Innovation Center for Cancer Medicine, Sun Yat-Sen University Cancer Center, Guangzhou, China; 3 Department of Radiology, Peking Union Medical College Hospital, Beijing, China; 4 Department of Radiology, The Third Affiliated Hospital of Sun Yat-sen University, Guangzhou, China

**Keywords:** Liver neoplasms, Haemangioendothelioma (epithelioid), Diagnostic imaging, Tomography (X-ray computed), Magnetic resonance imaging

## Abstract

**Objective:**

To improve the current imaging understanding of MRI or CT for hepatic epithelioid haemangioendothelioma (HEHE) to aid in its successful preoperative diagnosis.

**Methods:**

The imaging features of 15 patients (median age 38.6, range 20–71; 7 M/8 F) from eight institutions with pathologically confirmed HEHE were retrospectively analysed. Additionally, the CT/MR imaging features of 180 patients in 15 literature publications were collected, analysed and compared with our case series.

**Results:**

Fifteen patients underwent CT and MRI (n = 2), CT (n = 9) or MR (n = 8) scans. A total of 92.9% (13/14) of the patients were initially diagnosed with other lesions on imaging. A total of 86.7% (13/15) were multifocal. Nodules (11/15, 73.3%) were predominantly peripheral in distribution (12/15, 80.0%). Some cases were associated with hepatic capsular retraction (13/15, 86.7%), “target signs” (8/15, 53.3%) and “lollipop signs” (5/15, 33.3%). Peripheral enhancement of various shapes in the early phase with a progressive centripetal filling was the most common pattern of enhancement (12/15, 80.0%). Abnormal vascularity was seen in 50.7% (6/15) of the patients. Suspicious tumour thromboses in the inferior vena cava were seen in 3 (20.0%) of the patients. Two of the 15 patients (13.3%) had a history of smoking.

**Conclusions:**

HEHEs have common distinctive features, including multifocal lesions that are predominantly peripheral, “target signs”, “lollipop signs”, hepatic capsular retraction and peripheral enhancement of various shapes in the early phase with progressive centripetal filling. Additional aggressive imaging features that may be valuable clues to the diagnosis can be identified by CT or MRI.

**Supplementary Information:**

The online version contains supplementary material available at 10.1186/s13244-022-01344-y.

## Key points

- “Target signs” and “lollipop signs” should be recognised in imaging evaluation, raising the possibility of HEHE in the differential diagnosis.
- Abnormal blood vessels in tumours may have implications for imaging diagnosis.
- When HEHE is suspected, radiologists should be aware of tumour thrombi in the vena cava.
- HEHEs always present with a peripheral enhancement of various shapes with centripetal progressive filling.

**Supplementary Information:**

The online version contains supplementary material available at 10.1186/s13244-022-01344-y.

## Introduction

Hepatic epithelioid haemangioendothelioma (HEHE) is a rare vascular-derived tumour composed of epithelioid endothelial cells and dendritic cells or intermediate cells [1]. The 2019 WHO classification of tumours of the digestive system [2] classifies it as a malignant tumour, but it is now generally considered a tumour of low-moderate malignancy between hepatic cavernous haemangioma and angiosarcoma in terms of the clinical outcome [3–5]. Since Ishak et al. [6] first reported 32 cases in 1984, HEHE has gradually gained the attention of clinicians and pathologists. Because it is a rare tumour with an annual incidence of less than one per million, approximately 60–80% of HEHE tumours are misdiagnosed histopathologically [7]. Obtaining a preoperative diagnosis remains a challenge for both radiologists and referring physicians.

We collected 15 cases of pathologically confirmed HEHE from eight hospitals over a 10-year period from 2010 to 2020. An additional 180 cases were analysed in 15 articles from 2000 to 2021 to describe the imaging characteristics of HEHE. Through a literature review combined with these case analyses, we aim to gain an in-depth understanding of the clinical and imaging features of HEHE.

## Materials and methods

### Clinical HEHE cases

The institutional review board’s approval of eight hospitals was obtained for this retrospective study. Written informed consent was not required because the study involved a review of anonymised imaging only. The inclusion and exclusion criteria are presented in Additional file 1: Fig. S1. We obtained the imaging data (CT and MRI) and clinical information of the corresponding patients through the Picture Archiving and Communication System (PACS) and medical record system of the respective institutions. Fifteen patients from eight hospitals between the period of September 2012 and June 2020 were included in our study. Six patients underwent surgical resection, and nine underwent liver biopsy to obtain pathological specimens. All patients were finally diagnosed as having HEHE histopathologically.

### CT and MRI technique of the 15 HEHE cases

Given that the patients were extracted from a 10-year database of eight different hospitals, the specific CT and MR protocols varied considerably. We studied the CT/MRI sequences that are common to all patients, but there was no specific imaging protocol (e.g. manufacturers of contrast agents and machines, scan parameters). In our series, nine patients underwent dynamic triple-phase contrast-enhanced CT scans with intravenous iodinated contrast, eight patients underwent dynamic contrast-enhanced MR scans, and only two underwent both CT and MR scans. MR examinations were performed with 1.5 T/3.0 T scanners. We focused on the common sequences of unenhanced axial T1-weighted images (T1WI), axial/coronal fat-suppressed T2-weighted images (T2WI), diffusion-weighted images (DWI) and axial/coronal enhanced T1WI. Contrast-enhanced scans were performed on T1-weighted sequences with intravenous gadolinium contrast in two patients, and the arterial, portal, and equilibrium phases were obtained by breath holding (with a delay of approximately 3 min). Six patients underwent hepatocyte-specific imaging by gadoxetic acid contrast agents (also called gadolinium ethoxybenzyl diethylenetriamine pentaacetic acid, Gd-EOB-DTPA), and the hepatobiliary phase (HBP) was obtained 20 min after the injection.

### Clinical interpretation of the 15 HEHE cases

We retrospectively analysed sex, age, clinical symptoms and physical signs, past medical history, exposure history (including toxins, chemicals, drugs, tobacco and alcohol, etc.), liver function and tumour markers of the patients. The liver function parameters included γ-glutamyl transpeptidase (GGT), aspartate aminotransferase (AST), alanine aminotransferase (ALT), alkaline phosphatase (ALP) and cholinesterase (CHE). The tumour markers include alpha-fetoprotein (AFP), carcinoembryonic antigen (CEA), carbohydrate antigen 199 (CA 19–9) and carbohydrate antigen 125 (CA 125). All the indicators were considered abnormal if they were above or below the normal range.

### Image interpretation of the 15 HEHE cases

All original films were reviewed by two radiologists with 5 years and 19 years of experience. The final consensus was reached by group discussion when the opinions were inconsistent. The specific protocol of the radiological features is presented in Additional file 1: Table S1. The “target sign” of HEHE on imaging has been previously defined [8, 9], which is a tumour that shows a triple-ring or double-ring appearance on imaging. The “lollipop sign” is composed of a tumour and hepatic vein or portal vein around the tumour, which looks like a “lollipop” on contrast-enhanced imaging, as described by prior studies [9, 10]. For multifocal lesions, we examined the largest lesion or the most typical lesion and measured their maximum long-axis diameter. The concrete performance of four enhancement patterns is presented in Table S2. Pattern A is derived from our summary of previous research studies [11–14]. Pattern B was described by Sanduzzi-Zamparelli et al. [15]. Pattern C resembles the typical enhancement of hepatocellular carcinoma (HCC) [16]. Additionally, we also observed invasion of the main portal vein or hepatic vein and its branches, abnormalities in the background liver, and lesions in other organs. Additional imaging studies, such as ultrasound, angiography, and PET, were not the focus of this study.

### Literature review

We searched the databases PubMed and Google Scholar using the keywords “liver” or “livers” or “hepatic” or “hepatics” and “epithelioid haemangioendothelioma” in various combinations. The 15 articles published from 2000 to 2021 were case reports or case series with clinical and imaging characteristics. The inclusion and exclusion criteria are shown in Additional file 1: Fig. S2, which was jointly developed by all authors and were implemented by two of the authors.

### Statistical analysis

Analysis of the 15 patients was performed using IBM SPSS Statistics (version 25.0). Numerical variables were expressed as the mean ± standard deviation (SD) if normally distributed, while the median and interquartile range (IQR) were provided for nonnormally distributed data. Ordinal and categorical variables were expressed as numbers and percentages.

## Results

### Clinical findings and image findings of the 15 HEHE patients

The clinical and imaging findings of the 15 HEHE patients are presented in Table 1.

**Table 1:** Summary of clinical, histological and imaging characteristics of 15 patients

Male/female	7/8
Age	38.6 ± 14.4
Clinical presentation	
Asymptomatic	6/15 (40.0%)
Abdominal pain	8/15 (53.3%)
Weight loss	2/15 (13.3%)
Abdominal distension	2/15 (13.3%)
Others	3/15 (20.0%)
Previous medical history and exposure history	
Cirrhosis	3/15 (20.0%)
Long-term smoking	2/15 (13.3%)
HBV infection	2/15 (13.3%)
Surgical history	2/15 (13.3%)
Biliary system disease	2/15 (13.3%)
Others	4/15 (26.7%)
Tumour markers	
CA 125 ↑	3/15 (20.0%)
Liver enzymes	
ALT ↑	6/15 (40.0%)
GGT ↑	5/15 (33.3%)
ALP ↑	3/15 (20.0%)
AST ↑	3/15 (20.0%)
CHE ↓	1/15 (6.7%)
No. of CT	9
No. of MRI	8
No. of CT and MRI	2
Misdiagnosis by CT and MRI	13/14 (92.9%)
Pre-surgical imaging diagnosis by CT	8
Misdiagnosed as other tumours	8/8 (100%)
Metastases	3/8 (37.5%)
Intrahepatic cholangiocarcinoma	2/8 (25.0%)
Hepatocellular carcinoma	1/8 (12.5%)
Hepatic adenoma or focal nodular hyperplasia	1/8 (12.5%)
Unclear diagnosis	1/8 (12.5%)
Pre-surgical imaging diagnosis by MRI	7
Misdiagnosed as other tumours or lesions	6/7 (85.7%)
Unclear diagnosis	2/7 (28.5%)
Inflammatory lesions	2/7 (28.5%)
Metastases	1/7 (14.3%)
Haematolymphoid tumours	1/7 (14.3%)
Hepatic adenoma or focal nodular hyperplasia	1/7 (14.3%)
Hepatic epithelioid haemangioendothelioma	1/7 (14.3%)
Quantity	
Multifocal	13/15 (86.7%)
Unifocal	2/15 (13.3%)
Size	
Nodules	11/15 (73.3%)
Diffuse lesions	2/15 (13.3%)
Isolated masses	2/15 (13.3%)
The maximum diameter of the dominant tumour	36.0 (20.0, 70.0)
Coalescent	6/15 (40.0%)
Distribution	
Peripheral	8/15 (53.3%)
Peripheral + Central	4/15 (26.7%)
Diffuse	2/15 (13.3%)
Central	1/15 (6.7%)
Morphology	
Regular	9/15 (60.0%)
Irregular	6/15 (40.0%)
Boundaries	
Clear	11/15 (73.3%)
Less clear/unclear	4/15 (26.7%)
Pseudo-capsules	2/15 (13.3%)
Extrahepatic suspicious lesions	
Lungs	6/15 (40.0%)
Others	4/15 (26.7%)
Density on CT	
Hypodensity	8/9 (88.9%)
Isodensity	1/9 (11.1%)
Calcification	3/15 (20.0%)
T1WI on MRI	
Hypointensity	7/8 (87.5%)
Isointensity	1/8 (12.5%)
T2WI on MRI	
Hyperintensity	7/8 (87.5%)
Isointensity	1/8 (12.5%)
DWI on MRI	
Hyperintensity	7/8 (87.5%)
Isointensity	1/8 (12.5%)
Contrast enhancement on triple-phase	
Pattern A	13/15 (86.7%)
Pattern B	1/15 (6.7%)
Pattern C	1/15 (6.7%)
No. of the HBP	6
Homogeneous hypointensity	4/6 (66.7%)
Mixed hypointensity 1	1/6 (16.7%)
Mixed hypointensity 2	1/6 (16.7%)
Abnormal vascularity within tumours	6/15 (40.0%)
Target signs	8/15 (53.3%)
Lollipop signs	5/15 (33.3%)
Hepatic capsular retraction	13/15 (86.7%)
Portal vein invasion	5/15 (26.7%)
Hepatic veins invasion	2/15 (13.3%)
Tumour thromboses in the vena cava	3/15 (20.0%)

↑, elevate; ↓, decrease; *HBV* hepatitis B virus, *HAV* hepatitis A virus, *GGT* γ-glutamyl transpeptidase, *AST* aspartate aminotransferase, *ALT* alanine aminotransferase, *ALP* alkaline phosphatase, *CHE* cholinesterase, *CA125* carbohydrate antigen125; Pattern A enhancement, peripheral enhancement of various shapes on the arterial phase, with centripetal progressive filling accompanied by a decrease in signal intensity; Pattern B, mild enhancement on the arterial phase, with centripetal progressive filling on the next two phases along with gradually increasing intensification; Pattern C, marked enhancement on the arterial phase of the whole tumour, and washout occurred quickly in the next two phases. Mixed hypointensity 1, clearly hypointense in the centre and slightly low intensity in the peripheral ring; mixed hypointensity 2, hyperintense in the centre and hypointense in the peripheral ring

The 15 patients included 7 males and 8 females, with a mean age was 38.6 ± 14.4 years (range, 20–71 years).

**Fig. 1:**
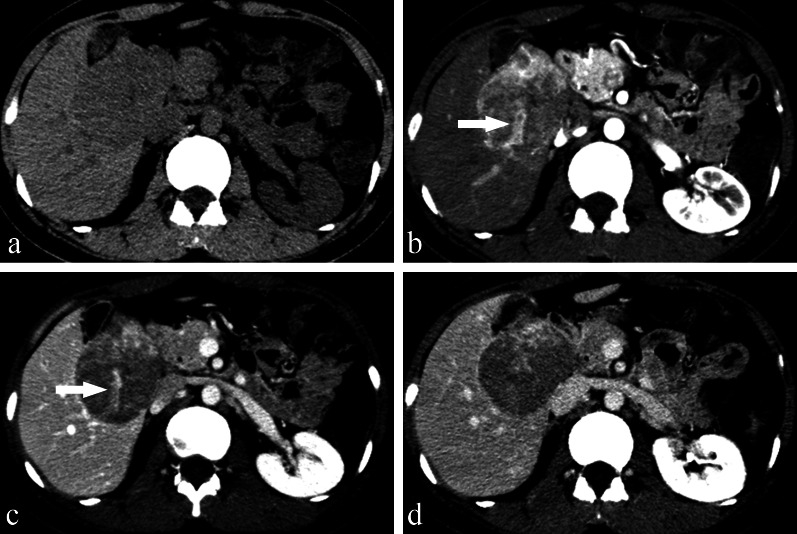
A 24-year-old female with pathologically diagnosed HEHE. **a** Axial unenhanced CT shows an isolated mass in the centre of the liver. Marked enhancement on the arterial phase, (**b**) of the whole tumour, and washout occurred quickly in the next two phases (**c**, **d**). Enlarged, persistently enhancing blood vessels are seen within the tumour (*arrows,* **b**, **c**)

**Fig. 2:**
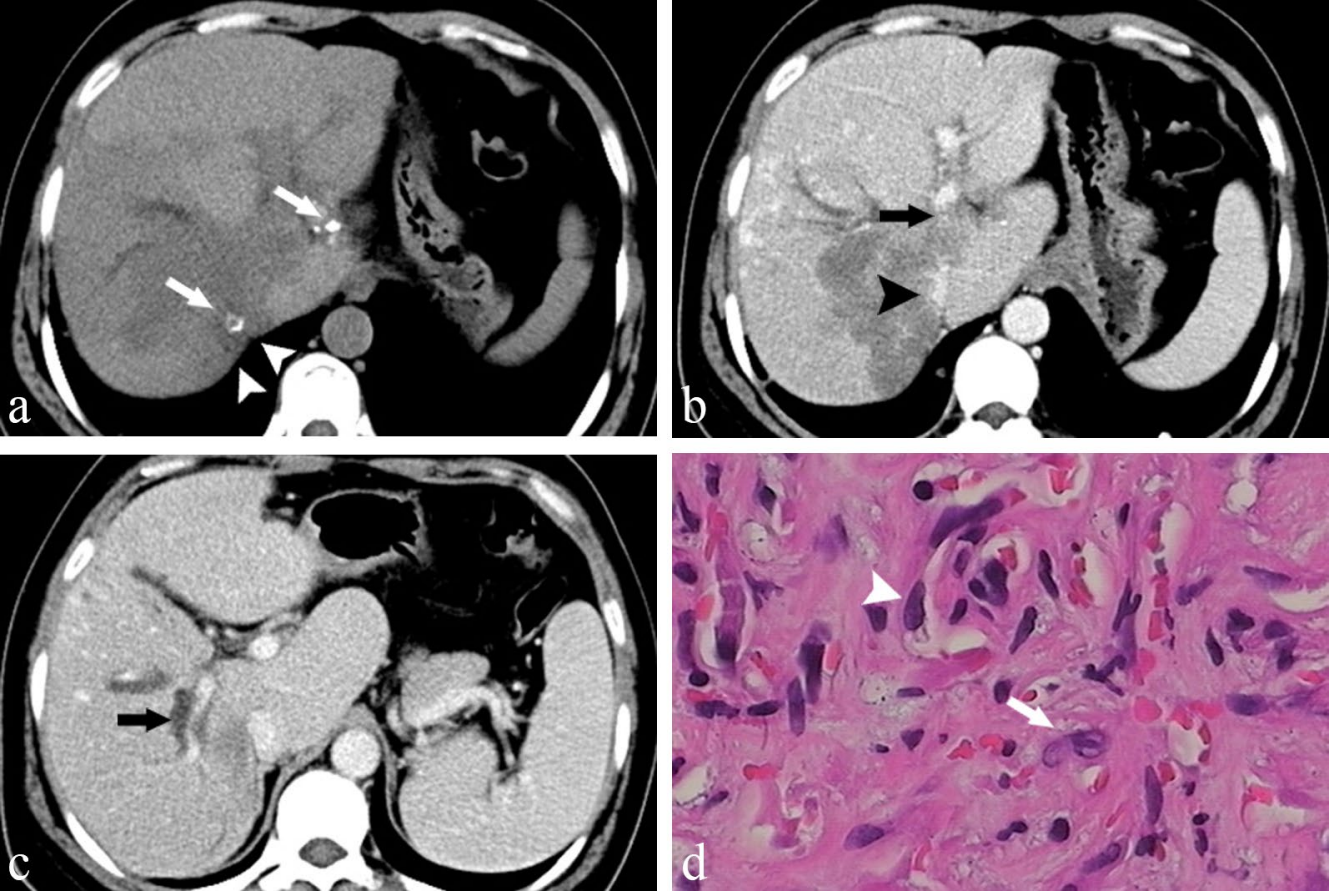
A 40-year-old female pathologically confirmed HEHE before the CT examination. **a** Axial unenhanced CT illustrates nodules coalesced into a mass with ill-defined borders; hepatic capsular retraction (*arrowheads*) and calcification inside the tumour (*arrows*). Axial portal venous phase contrast-enhanced CT demonstrates left portal vein invasion (*arrow,* **b**), inferior vena cava invasion (*arrowhead,* **b**) and right portal vein invasion (*arrow,* **c**). **d** The histopathological section shows some dendritic (*arrowhead*) and endothelial cells (arrows) making primitive vascular structures with red blood cells contained

**Fig. 3:**
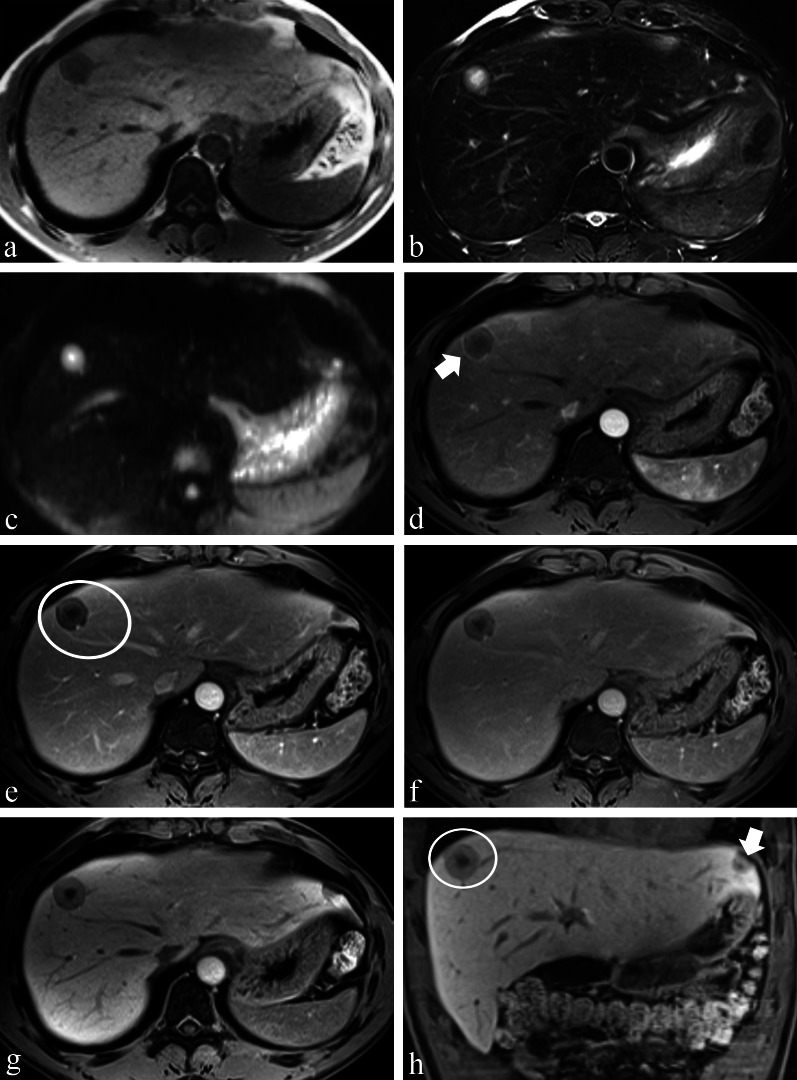
A 42-year-old male with pathologically confirmed HEHE, which was initially diagnosed as metastases. **a** Axial T1WI illustrates a hypointense tumour in segment 8 of the liver; **b**, **c** the “target sign” was composed of slight peripheral hyperintensity and bright central hyperintensity on axial T2WI and DWI. **d**–**h** Contrast-enhanced MRI after administration of gadoxetic acid shows enhancement pattern A of the tumour. **d** The axial arterial phase shows a peripheral thin ring enhancement (*arrow*); the nodule showed the typical “target sign” on all phases after enhancement (*circle,* **h**), and the “lollipop sign” on the portal venous phase (*circle,* **e**). There was another lesion with hepatic capsular retraction (*arrow*) on the coronal HBP (**h**)

**Fig. 4:**
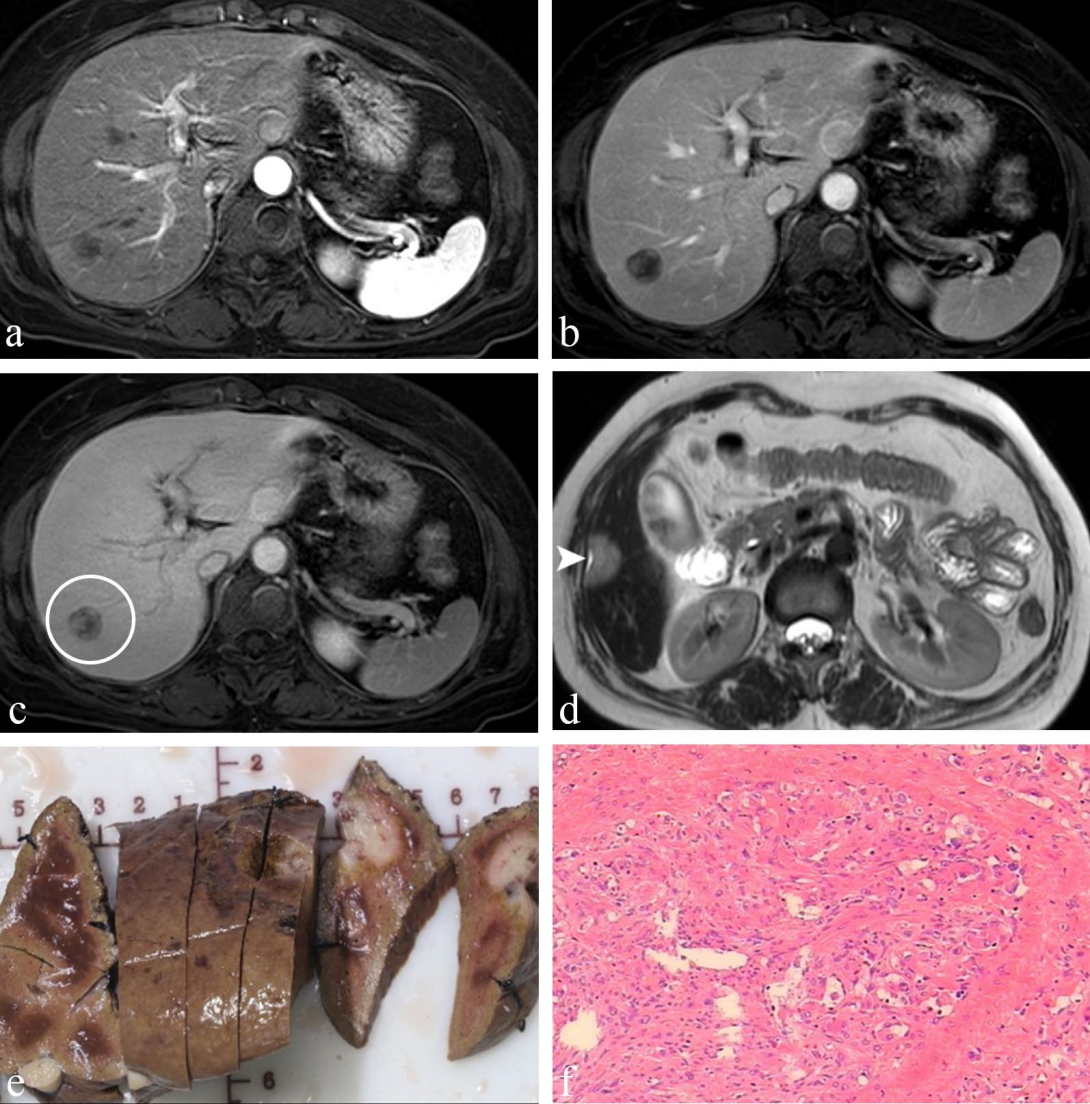
A 53-year-old woman was diagnosed with HEHE on both imaging and pathology. **a**–**c** On axial contrast-enhanced MRI, the tumour in segment 6 of the liver represents a centripetal progressive filling with a decrease in signal intensity and finally shows a targetoid appearance on the equilibrium phase (*arrow,* **c**). There was another lesion with hepatic capsular retraction (*arrowhead*) on axial T2WI (**d**). **e** Gross solid specimen after surgery shows multifocal lesions located in the periphery of the liver. **f** Hematoxylin–eosin stain reveals that tumours are mainly composed of eosinophilic epithelioid cells arranged in sheet-like structures

**Fig. 5:**
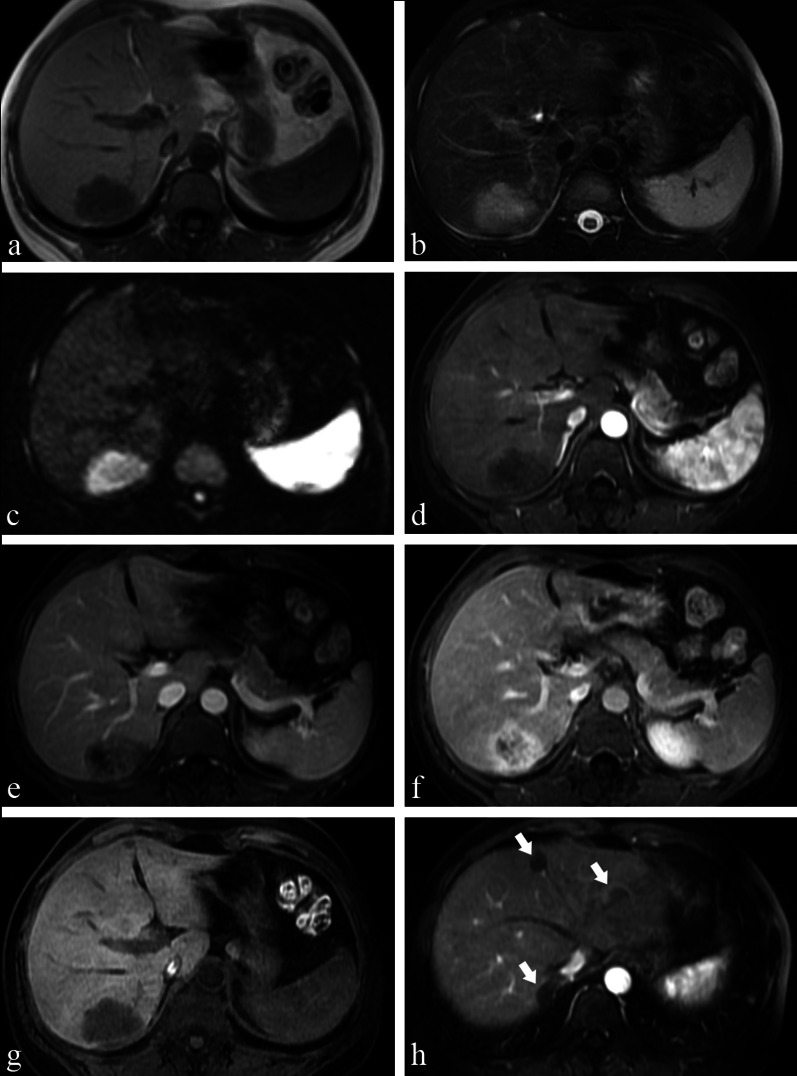
A 36-year-old female with pathologically diagnosed HEHE. Axial unenhanced T1-weighted MRI (**a**) demonstrates a hypointense tumour in segment 6 of the liver, which is hyperintense on axial fat-suppressed T2WI (**b**) and DWI (**c**). On contrast-enhanced images of three phases (**d**, **f**), the tumour presented enhancement pattern A, with peripheral irregular enhancement on arterial phase (**d**), with centripetal progressive filling on the portal venous phase and (**e**) equilibrium phase (**f**) along with gradually increasing intensification, and a “lollipop sign” can be seen on portal venous phase. The tumour manifested as a homogeneous hypo-intensity on the HBP (**g**). There were other lesions (arrows) in the upper part of the liver on the arterial phase (**h**) with pathologically proven epithelioid haemangioendothelioma

**Fig. 6:**
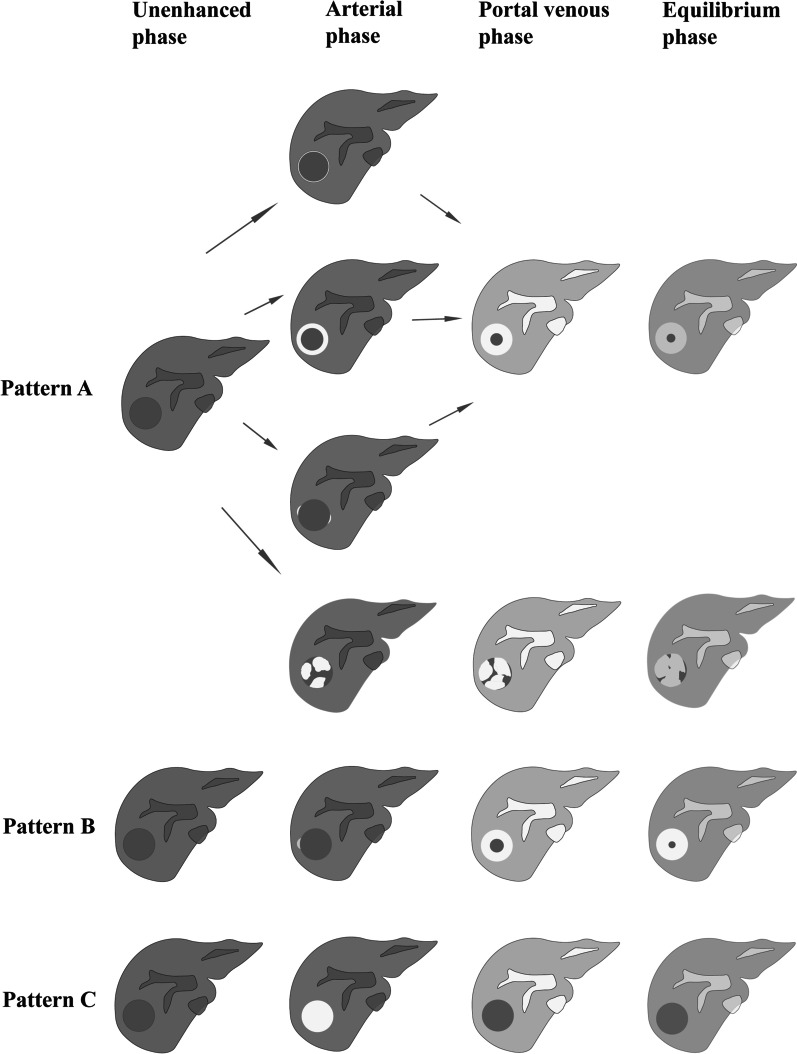
The three patterns of contrast enhancement on triple-phase CT or MRI scans. Grey nodules represent tumour entities. The white colour represents an enhancement of the tumours, and as the whiteness increases, so does the degree of enhancement

Thirteen patients were initially diagnosed as having intrahepatic cholangiocarcinoma (ICC), metastases and others (see Table 1), and there was a high misdiagnosis rate of 92.9% (13/14). Imaging of one of the 15 patients was performed after a needle biopsy; therefore, for this patient, the data we collected were only used to analyse clinical and imaging features and were not included in the misdiagnosis rate. Thirteen patients had multifocal lesions, and two had unifocal lesions (Fig. 1 and Additional file 1: Fig. S3). The majority of the tumours (12 of the 15 patients) were peripheral in distribution. One tumour was located in the centre (Fig. 1), and two patients had a diffuse distribution of lesions throughout the liver (Additional file 1: Fig. S4). Thirteen patients had hepatic capsular retraction (Figs. 2, 3, 4, Additional file 1: Figs. S5 and S6). The “target sign” was seen in eight of the patients (Figs. 3, 4, Additional file 1: Figs. S3 and S5), and the “lollipop sign” was seen in five of the patients (Fig. 3 and Additional file 1: Fig. S5). Lesions in other organs were suspected metastases of HEHEs (Additional file 1: Figs. S4 and S7). On dynamic contrast-enhanced imaging, three patterns of enhancement were observed (Additional file 1: Table S2, Figs. 5, 6). The most common pattern, which was a peripheral enhancement of various shapes on the arterial phase, with centripetal progressive filling accompanied by a decrease in signal intensity, appeared in 13 patients (Figs. 2, 3, 4 and Additional file 1: Figs. S3–S7). Abnormal vascular structures within tumours were seen in 6 patients (Figs. 1, 2, 5, Additional file 1: S3 and S5). Portal vein branches were involved in five of the patients (Fig. 2), of which two patients had hepatic vein invasion. Tumour thromboses in the vena cava were seen in 3 of the patients (Figs. 2, Additional file 1: S6 and S7).

### Clinical data and image findings of literature review

The clinical and general imaging findings of the 15 articles are listed in Table 2. The density and signal characteristics on the imaging of 169 patients are summarised in Table 3. All 15 articles [8, 11–13, 15, 17–26] included four case reports and 11 case series or retrospective cohort studies. A total of 180 patients diagnosed with HEHE have been reported, with a male-to-female ratio of 3:5 (67:113). Four articles [13, 20, 23, 26] studied all of the lesions in each patient’s liver, and the remaining were studied on a patient-unit basis.

**Table 2:** Summary of clinical and general imaging characteristics of literature review

Cases of the 15 studies	180
Male/female	3/5 (67/113)
Mean/median age, years (11 studies)	37.5–51.9
Clinical Presentation (11 articles, 78 cases)	
Asymptomatic	37/78 (47.4%)
Abdominal pain	17/78 (22.8%)
Weight loss	8/78 (10.3%)
Abdominal distension	5/78 (6.4%)
Fatigue	5/78 (6.4%)
Others	6/78 (7.7%)
Past medical history and exposure history (13 articles, 161 cases)	
HBV infection	3/161 (1.9%)
HCV infection	3/161 (1.9%)
Cirrhosis	2/161 (1.2%)
Surgical history	2/161 (1.2%)
Liver steatosis	2/161 (1.2%)
Others	3/161 (1.9%)
Tumour markers (10 articles, 51 cases)	
CEA ↑	2/51 (3.9%)
CA 125 ↑	1/51 (2.0%)
AFP ↑	1/51 (2.0%)
Liver enzymes (8 articles, 39 cases)	
ALP ↑	4/39 (10.3%)
GGT ↑	3/39 (7.6%)
AST ↑	3/39 (7.6%)
ALT ↑	1/39 (2.6%)
Cases with imaging studies	169
No. of CT	121 (11 articles)
No. of MRI	103 (15 articles)
Gd-EOB-DTPA	20 (4 articles)
Quantity	
Multifocal	143/169 (83.4%)
Unifocal	26/169 (15.4%)
Size	
Nodules	139/169 (82.2%)
Diffuse lesions	4/169 (2.3%)
Coalescent	66/141 (46.8%)
Calcification (9 articles, 119 cases)	23/119 (19.3%)
Extrahepatic HEHE (8 articles, 59 cases)	
Lungs	17/59 (28.8%)
Bone	5/59 (8.4%)
Peritoneum	1/59 (1.7%)
Portal vein/hepatic vein invasion (9 articles, 118 cases)	46/118 (3.9%)

*↑* elevate; *GGT* γ-glutamyl transpeptidase, *AST* aspartate aminotransferase, *ALT* alanine aminotransferase, *ALP* alkaline phosphatase, *CEA* carcinoembryonic antigen, *CA125* carbohydrate antigen125; *AFP* alpha-fetoprotein. HBV infection, hepatitis B virus infection; HCV infection, hepatitis C virus infection

**Table 3:** Density and signal characteristics on imaging of literature review

Author, year	No. of Cases	CT		MRI						Target sign	Lollipop sign	Hepatic capsular retraction	Notes
	CT/MRI (HBP)	Density	Enhancement	T1WI	T2WI		DWI	Enhancement					
Semelka et al. 2018 ^[[8]]^	0/13(0)	−	−	Hypo		Hyper	None	A: Peripheral ring enhancement (12/13); V, E: Progressive centripetal enhancement (9/13)	9/13		None	8/13	−
Ganeshan et al. 2020 ^[[11]]^	67/30 (7)	Hypo	A: Peripheral ring enhancement (21/64); V: Target-like appearance (46/67)	None		Heterogeneous Hyper (29/30)	Heterogeneous Hyper (11/30)	Same as CT enhancement HBP: Homogeneous Hypointensity (3/7) Target-like appearance (4/7)	46/67		20/67	54/67	−
Giardino et al. 2016 ^[[12]]^	0/7(0)	−	−	Moderate Hypo		Moderate Hyper	None	A: Peripheral ring enhancement (3/7); V: Centripetal progressive filling (7/7); E: Delayed enhancement (5/7)	5/7		None	4/7	−
Zhou et al. 2015 ^[[13]]^	11/5(0)	Hypo	A: Mild uniform enhancement; V, D: No progressive enhancement (223/312)	Hypo		Heterogeneous Hyper	None	Same as CT enhancement		None	6/11	6/11	A total of 312 nodules were detected in 11 patients
Sanduzzi-Zamparelli et al. 2020 ^[[15]]^	6/7(0)	Hypo	Progressive enhancement with gradually increasing enhancement (6/13)	Hypo		Iso/Hyper	None	Same as CT enhancement		None	None	2/13	Imaging analysis was performed in only 13 of the 24 patients
Leonardou et al. 2002 ^[[17]]^	0/1(0)	−	−	Moderate Hypo		Moderate Hyper	None	Peripheral ring enhancement		None	None	None	−
Thin et al. 2010 ^[[18]]^	5/4(0)	Hypo	Delayed enhancement	Moderate Hypo		Moderate Hyper	None	Delayed enhancement		2/5	None	3/5	−
Azzam et al. 2012 ^[[19]]^	1/1(0)	Hypo	Mild or no enhancement in early stages with progressive filling	Hypo		Heterogeneous Hyper	None	Obvious ring enhancement on the arterial phase with centripetal progressive filling and decreasing intensity		1	−	1	−
Gan et al. 2016^[[20]]^	7/9(0)	Hypo	Centripetal enhancement	Hypo		Heterogeneous Hyper	Heterogeneous Hyper	Progressive peripheral ring enhancement (160/229)		141/229	10/229	60/229	A total of 229 lesions were detected in 14 patients
Galletto et al. 2016 ^[[21]]^	1/1(0)	Hypo	A: No significant enhancement; V: Mild peripheral enhancement	Hypo		Heterogeneous Hyper	Heterogeneous Hyper	Mild, Heterogeneous and progressive enhancement		1	−	1	−
Paolantonio et al. 2014 ^[[22]]^	0/11(8)	−	−	Hypo		Heterogeneous Hyper	Heterogeneous Hyper	Mild or Peripheral ring enhancement (7/11). HBP: Homogeneous Hypointensity (5/8), Target-like appearance (3/8)		7/11	None	5/11	−
Chen et al. 2011 ^[[23]]^	7/5(0)	Hypo (6/7) Hyper (1/7)	None	Hypo (4/5); Iso (1/5)		Hyper (4/5), Iso/Hypo (1/5)	Hype (2/2)	A, V, E: Mild, ring or irregular progressive enhancement with lower intensification than the liver parenchyma; D: (130–142 s) The intensification of tumours is higher than the liver parenchyma (4/5)		CT (7/74); MRI (27/28)	None	None	7 cases with 74 nodules detected by CT and 5 cases with 28 nodules detected by MRI
Hsieh et al. 2010 ^[[24]]^	5/1(0)	None	None	None		None	None	Peripheral ring enhancement, and progressive centripetal enhancement (5/6)		None	None	2/6	−
Kim et al. 2015 ^[[25]]^	10/7(5)	Hypo	Progressive enhancement with mild enhancement on the arterial phase (7/7)	Hypo		Hyper	None	A, V, E: Mild, circumferential, centripetal enhancement (7/7) HBP: Homogeneous Hypointensity (3/5), Target-like appearance (2/7)		2/10	None	5/10	−
Fan et al. 2020 ^[[26]]^	1/1(0)	Hypo	A: Patchy enhancement; V: Homogeneous enhancement	Hypo		Slightly Hyper	Hype	Same as CT enhancement		1	−	−	A total of 27 nodules were detected

− , negative, *A* arterial phase, *V* portal venous phase, *E* equilibrium phase, *D* delay pattern, *HBP* hepatobiliary phase, *Hypo* hypodensity/intensity, *Iso* isodensity/intensity, *Hyper* hyperdensity/intensity

## Discussion

### Clinical features of HEHE

The 15 studies’ review shows that HEHE is more commonly seen in women and has a male-to-female ratio of 3:5. The median or the average age of the patients ranged from 37.5 to 51.9 years. The aetiology of HEHE is still unclear but may be related to the use of oral contraceptives or hormonal drugs, alcohol consumption, Crohn’s disease, hepatitis virus and exposure to vinyl chloride or asbestos [4, 7, 27, 28]. Notably, 13.3% of our 15 patients had a history of smoking, which has not been reported in other articles. The patients with HEHE are always asymptomatic, and of those patients with symptoms, most of them presented with abdominal pain [17–21]. In addition, they are often associated with abnormal laboratory tests, such as ALT, AST, and GGT, but tumour markers such as AFP, CEA and CA 19–9 are usually within the normal range [21, 24]. Uncommonly, in our series, 20.0% of the patients showed increased CA 125. Epithelioid haemangioendothelioma was found in organs other than the liver, according to literature publications [25, 26]. Similarly, in our cohort, extrahepatic suspicious lesions were found in 46.7% of the patients, 40.0% of which were in the lungs. Unfortunately, only one pulmonary suspicious lesion was pathologically confirmed as epithelioid haemangioendothelioma.

### Imaging findings with pathologic correlation

In our series, 92.9% of the patients diagnosed as having HEHE were initially diagnosed as having other hepatic diseases by CT and MRI, and the final diagnosis mainly depended on the histopathological findings. However, with the retrospective analysis and evaluation of the 15 patients and in combination with a literature review, we identified several imaging features that may indicate and support the diagnosis of HEHE. By reviewing the relevant literature, we analysed the correlation between these imaging features and pathology, as follows:

1. Pathologically, HEHE is composed of epithelioid endothelial cells and dendritic cells scattered in the mucus matrix within hyaluronic acid [29]. In the early stage, tumour cells are confined to hepatic sinusoids or distal small veins, with a pseudopolypoid or small nodular appearance [1, 6]. Correspondingly, HEHEs potentially appear as multifocal nodules of small sizes with regular morphology and well-defined borders, without pseudo-capsules, and are located in the periphery of the liver on imaging [11, 12]. The tumour increases in size in the late stages, sometimes leading the nodules to coalesce into a mass with ill-defined borders [15, 20]. This may result from the growth of tumour cells, which infiltrate pre-existing acini and small blood vessels, destroy basement membranes and then invade the surrounding liver sinuses [6, 30]. Of our 15 patients, 86.7% had the multifocal disease, and 40.0% of the nodules had confluence. In 13.3% of the patients, diffuse lesions were distributed throughout the liver, as reported in four patients in three literature publications [18, 23, 24]. A total of 80.0% of the patients had a predominantly peripheral distribution. All of the above findings were consistent with the pathological studies.
2. On unenhanced CT, the solid component of the tumour appears as low density. On unenhanced MRI, tumours show hypointensity on T1WI and hyperintensity on T2WI and DWI [22, 23]. As the tumour grows, HEHE gradually showed heterogeneous appearances. Prior reports showed that this was pathologically caused by degenerative changes in the tumours, including sclerotic, necrosed, and/or calcified [31, 32]. Tumour calcifications were seen in 20.0% of our patients, which is almost consistent with the literature review that showed these findings in 19.3% of patients.
3. The “target sign” is a round overt hypodense area surrounded by a slightly hypodense ring with uniform thickness on CT [30, 31]. On T2WI and DWI, it also shows distinct hyperintensity in the centre with a peripheral ring of slight hyperintensity and a thin ring of hypointensity in the outermost layer [30, 31]. Pathology confirmed that the core of the target was myxoid degeneration or cystic necrosis of the fibrous matrix in the centre of the tumour, the middle layer was the cell proliferation layer, and the outermost layer was composed of an avascular region between the tumour cells and normal liver parenchyma [9]. This sign appeared in 53.3% of the 15 patients. Chen et al. [23] found that the detection rate of CT for the “target sign” (9.5%) was significantly lower than that of MRI (96.4%). Likewise, in our 15 patients, the “target sign” was detected on CT (11.1%) and MRI (87.5%).
4. In previous studies, the “lollipop sign” is considered to be the most specific sign of HEHE, which is pathologically caused by occlusion or narrowing of the hepatic vein or portal vein around tumours [9, 10, 13]. The tumour is similar to the body of a lollipop, and the peripheral vein represents the lollipop stick [6, 30]. The “lollipop sign” was detected in 33.3% of the patients; it had a CT detection rate of 22.2% and an MRI detection rate of 50.0% in our 15 patients.
5. Hepatic capsular retraction occurred in 86.7% of the 15 patients. According to reports in pathology studies [8, 33, 34], tumour cells grow along the vascular lumen and infiltrate into the hepatic sinusoids, resulting in atrophy of hepatocytes and destruction or collapse of the liver plate. Moreover, the increased fibrous tissue within the tumour pulling on the surrounding liver tissue or degeneration of the fibrous matrix results in the collapse of the surrounding liver tissue, which may cause retraction of the hepatic capsule [8, 33, 34]. The hepatic capsular retraction occurred in 86.7% of the patients, and this is higher than the 62.3% that had been reported by 12 literature publications.
6. The enhancement pattern in HEHE has been proven to depend on the distribution of tumour cells, fibrous tissue and its degeneration degree, and tumour vascularity [19, 20, 25]. In the early stage, because the liver acini and blood vessels are not involved by tumour cells, only mild peripheral enhancement may be seen on contrast images. With the progressive fibrosis of the myxoid matrix from the centre of the tumour, HEHEs perhaps showed progressive centripetal enhancement accompanied by a decrease in signal intensity [19–21]. In addition, HEHE mainly showed hypointensity and rarely heterogeneous intensity during the HBP [8, 22]. Unusually, one of the 15 patients showed a washout enhancement pattern. From the imaging performance of this patient, we speculate that this pattern may be caused by the tumour invading the vessels in the hilar region, resulting in an arteriovenous fistula, which may mask the true enhancement pattern of the tumour. Additionally, another patient showed a progressive enhancement pattern with gradually increasing intensification, which is different from the previous studies.
7. Pathological studies revealed that the growth of the tumour cells, especially epithelioid cells, is characterised by crawling towards the hepatic hilum along distal small veins [6, 30]. The invasion of the hepatic vein or portal vein was seen in 3.9% of 9 literature publications and 26.7% of the 15 patients. We also found suspicious tumour thromboses in the vena cava in 20.0% of the patients, which had not been reported in other literature publications. Combined with the above pathological literature, we speculate this may be caused by tumour cells continuing to crawl along the hepatic vein. Moreover, abnormal vascular structures within tumours were seen in 40.0% of the patients. In pathology, these vessels may be tumour-supplying arteries, draining veins or neovascularisation of tumour cells [1, 26].

### Differential diagnosis

Makhlouf et al. [4] reported that 60–80% of HEHE patients were initially misdiagnosed in histopathology, while the misdiagnosis rate was even higher on preoperative imaging and reached 92.9% in our 15 patients. HEHE is often initially diagnosed as common liver tumours, such as ICC, metastases and HCC.

*ICC* progressive enhancement in ICC is most obvious during the delayed phase at 4–6 min after the injection of the contrast agent, whereas the maximum intensification of HEHE was observed approximately three minutes following the contrast injection in most of our 15 patients [35]. In addition, the typical imaging features of ICC are lobulated tumours, with ill-defined boundaries, peripheral dilated bile ducts and sub-tumours; the prevalent age of ICC is 55–75 years old; and ICC is more common in men, is associated with an increase in CA 19-9 and is usually accompanied by peripheral lymph node metastasis [36–38]. All of the above is helpful in distinguishing it from HEHE.

*Metastases* The “bull’s-eye sign” of metastases is similar to the “target sign” of HEHE in terms of pathology translating into a similar imaging pattern [39, 40]. However, in the early phase of enhanced MRI, there is an obvious perilesional enhancement around the “bull’s-eye sign”, which may be caused by dilatation of the surrounding hepatic sinusoids and infiltration of liver parenchyma by inflammatory cells [39]. Metastases mostly originate from primary foci such as colorectal cancer, pancreatic cancer and lung cancer. Therefore, finding the primary tumour is the key to distinguishing the two diseases. In addition, metastatic tumours, like their primary focus, may be accompanied by an increase in the levels of tumour markers (e.g. CAE, CA 19-9) [41, 42].

*HCC* The typical imaging findings of HCC are mostly solitary lesions with ill-defined borders, bulging liver capsules around the tumour, portal vein thrombosis and tumour pseudo-capsules on enhanced imaging [16]. HCC is dominant in men over 45 years old, who have viral hepatitis and elevated AFP, and these factors can help to confirm its diagnosis [16, 43].

There are several limitations in our study. This study was a retrospective study and had inherent shortcomings. The CT and MRI machine models and contrast agents used were different, which may result in differences in the imaging techniques, sensitivity and specificity. Besides, the number of patients is not sufficient for a more complex statistical analysis. For the literature review, first, due to the different focuses of the research in each article, features we studied were not covered in every article. Next, the description or definition of the same symptom is not necessarily the same in each article.

In summary, the main imaging features of HEHE (such as the “target signs”, the “lollipop signs”, hepatic capsule retraction, peripheral distribution and progressive centripetal enhancement) should be kept in mind when analysing liver tumours, mainly in the younger population. In addition, we also need to pay attention to a washout enhancement pattern, a progressive enhancement pattern with increasing intensification, metastatic lesions of other organs, invasion of the hepatic vein or portal vein, suspicious tumour thrombi in the vena cava, etc.

## Supplementary Information

**Additional file 1.** Inclusion/exclusion criteria; supplementary tables and figures.

## Data Availability

The datasets used and analysed during the current study are available from the corresponding author upon reasonable request.

## Abbreviations

CT: Computed tomography
HCC: Hepatocellular carcinoma
HEHE: Hepatic epithelioid haemangioendothelioma
ICC: Cholangiocarcinoma
MRI: Magnetic resonance imaging
