# Supplementary material for: CT and MRI features of hepatic epithelioid haemangioendothelioma: a multi-institutional retrospective analysis of 15 cases and a literature review
Source: Insights Imaging. 2023 Jan 5;14:2. doi: 10.1186/s13244-022-01344-y (PMC9813315; doi:10.1186/s13244-022-01344-y)
Supplement: Supplementary file 1 — Additional file 1. Inclusion/exclusion criteria; supplementary tables and figures. [file 13244_2022_1344_MOESM1_ESM.pdf]

ELECTRONIC SUPPLEMENTARY MATERIAL

CT and MRI features of hepatic epithelioid haemangioendothelioma: A multi-institutional retrospective analysis of 15 cases and a literature review

Figure S1

The study flowchart of recruited patients.

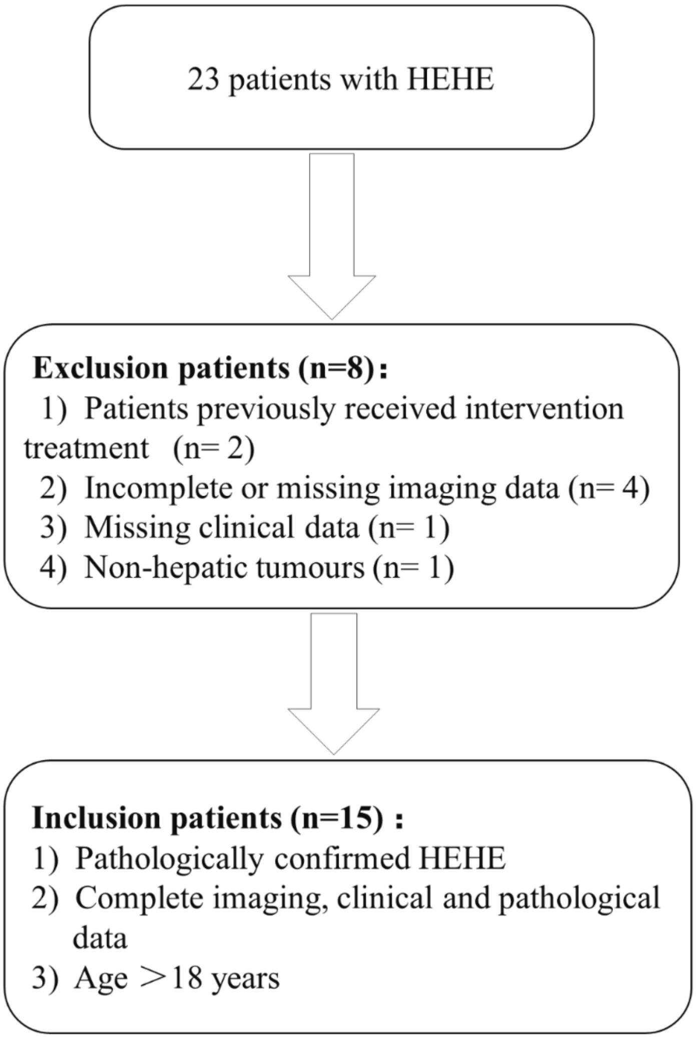

Table S1 Definitions of radiological features assessed on CT/MR images of patients with HEHE.

Features	Regulation	Standard
Size	Tiny nodules	≤ 10 mm
	Nodule	≤ 30 mm
	Mass	＞30 mm
Number	Unifocal/ Isolated	＜2
	Multifocal	≥ 2
Nodule fusion	With	–
	Without	–
Morphology	Regular	Round-like or oval-like
	Irregular	Other shapes
Margins	Clear	On unenhanced CT or MRI, the density or signal is significantly different from the surrounding liver parenchyma
	Unclear	–
Pseudo-capsules	With	A circular linear structure around tumours
	Without	–
Localization	Peripheral	Within 30 mm of the hepatic capsule
	Central	Beyond 30 mm of the hepatic capsule

Density/intensity	Hypo-dense/intense	
	Iso-dense/intense	Relative to the surrounding liver
	Hyper-dense/intense	
Calcification	With	≥100 HU
	Without	＜100 HU
Hepatic capsular retraction	With	A depressed or flattened hepatic capsule
	Without	−
Target sign	With	A triple-ring or double-ring appearance
	Without	−
Lollipop sign	With	A lollipop appearance
	Without	−
Enhancement characteristics	Degree	Relative to the surrounding liver
	Pattern	As described in supplementary table S2

Note. Hounsfield unit (HU) is a dimensionless unit universally used in computed tomography (CT) scanning to express CT numbers in a standardized form. −, none.

Table S2 The concrete performance of four enhancement patterns after injection of contrast media of our study.

Pattern	The concrete performance	NO. of cases
**A**	Peripheral thin rim, thick rim, nodular or irregular (few areas of patchy enhancement) enhancement on the arterial phase, with centripetal progressive filling accompanied by a decrease in signal intensity	12/15
**B**	Mild enhancement on the arterial phase, with centripetal progressive filling on the next two phases along with gradually increasing intensification	1/15
**C**	Marked enhancement on the arterial phase of the whole tumour and washout happened quickly on the next two phases	1/15

Figure S2

The flowchart of literature screening.

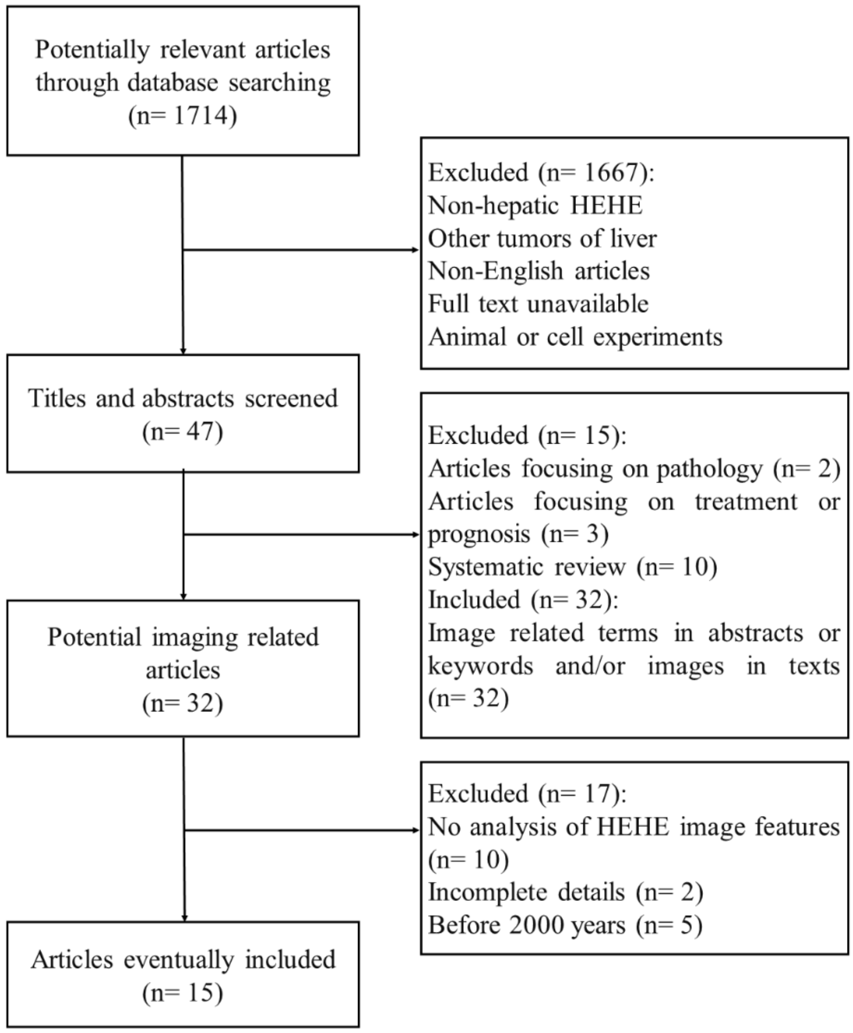

Figure S3

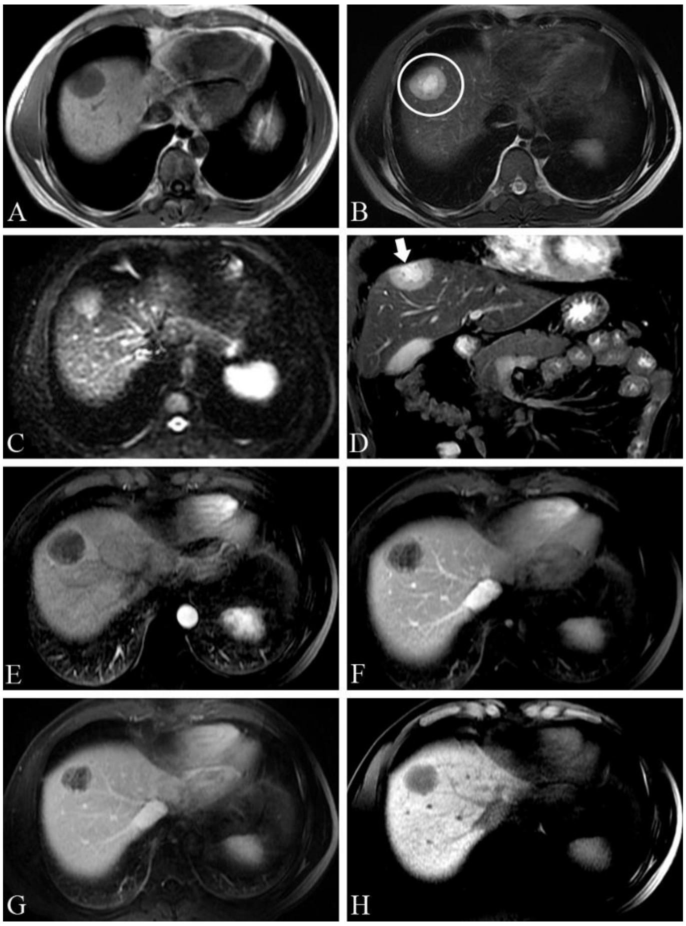

Figure S3. MR images of a 40-year-old male show an isolated tumour of approximately 36 mm in diameter. Axial unenhanced T1-weighted MRI (**A**) illustrates a hypointense tumour, with a “target sign” (*circle*) on axial fat-suppressed T2WI (**B**) and slight hyperintensity on DWI (**C**); coronal fat-suppressed T2-weighted MRI (**D**) indicates the sign of hepatic capsular retraction (*arrow*). On contrast-enhanced MRI (**E-H**) with gadoxetic acid contrast agent: the tumour presented enhancement pattern A, with a mild thin ring enhancement on the axial arterial phase (**E**), and centripetal progressive filling on the next two phases, with grid-like small blood vessels inside the tumour (**F**,

G); there is no contrast agent uptake in the tumour on HBP (**H**).

Figure S4

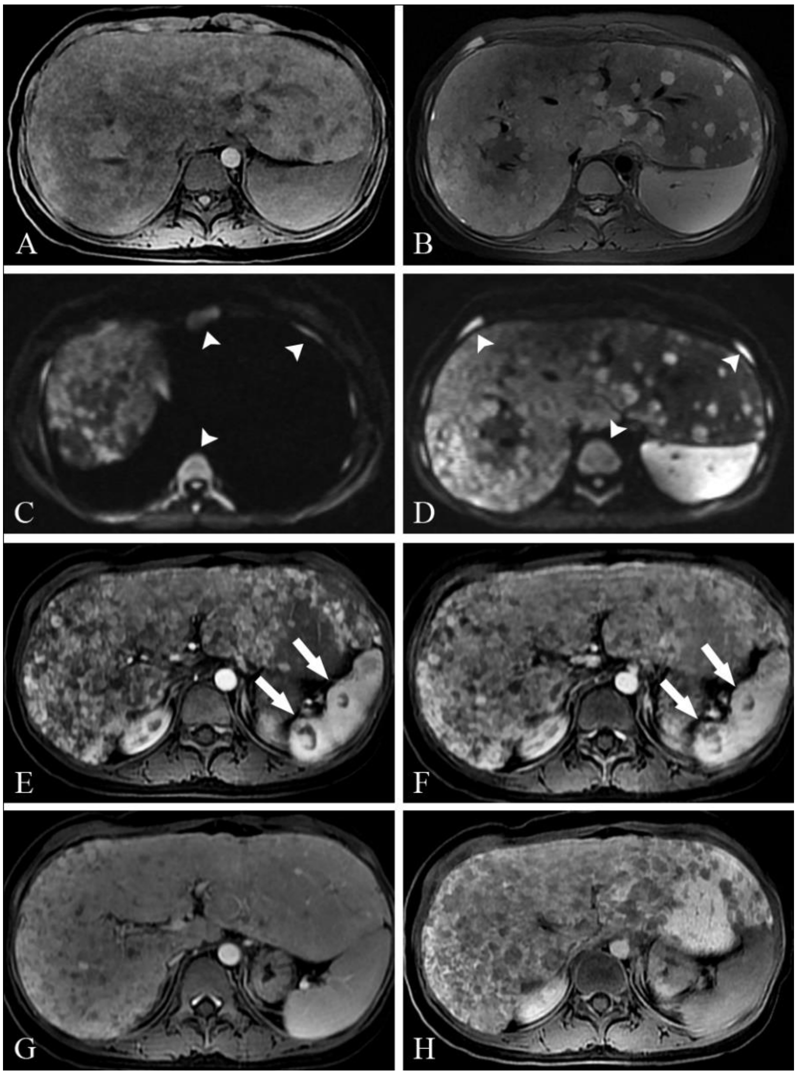

Figure S4. MRI of a 20-year-old female who was initially diagnosed with haematolymphoid tumours. (**A**) Axial unenhanced T1-weighted MRI shows diffuse multifocal lesions with hypointensity spreading all over the liver; (**B**) axial fat-suppressed T2-weighted MRI reveals these hyperintense nodules; (**C**) and (**D**) axial diffusion-weighted MRI displays tumours with a ring or round hyperintensity and abnormal hyperintense lesions in the sternum, vertebrae and ribs (*arrowheads*). (**E**-**H**)

On contrast-enhanced images, the tumours showed enhancement pattern A, thick rim enhancement on the arterial phase (**E**), with progressive filling on the portal venous and equilibrium phases (**F-G**), and no contrast-agent retention on the HBP of MRI. Suspicious lesions were found in the spleen (*arrows,* **E** *and* **F**).

Figure S5

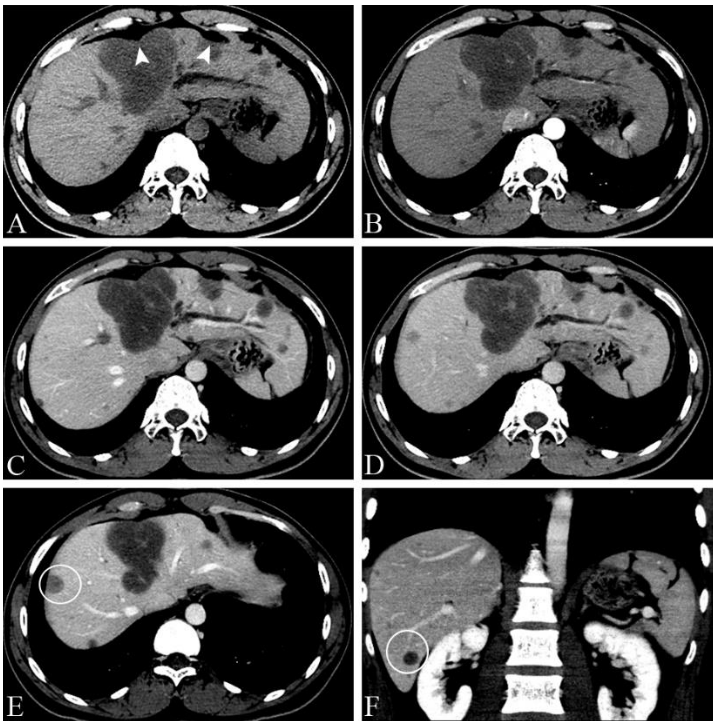

Figure S5. CT of a 26-year-old man who was initially diagnosed as having an intrahepatic cholangiocarcinoma. (**A**) Axial unenhanced CT shows multifocal hypodense nodules in the liver that are partially coalesced into a mass, and the sign of hepatic capsular retraction (*arrowheads*); (**B**) axial arterial phase contrast-enhanced CT demonstrates moderate heterogeneous enhancement in these tumours, and slightly filled in portal venous phase (**C**) and equilibrium phase (**D**), which presents in enhancement pattern A. The "target sign" located in the upper right anterior lobe on

the portal venous phase (*circle*) (**E**); the "lollipop sign" in segment 6 of the liver (*circle*) appeared on the coronal portal venous phase (**F**).

Figure S6

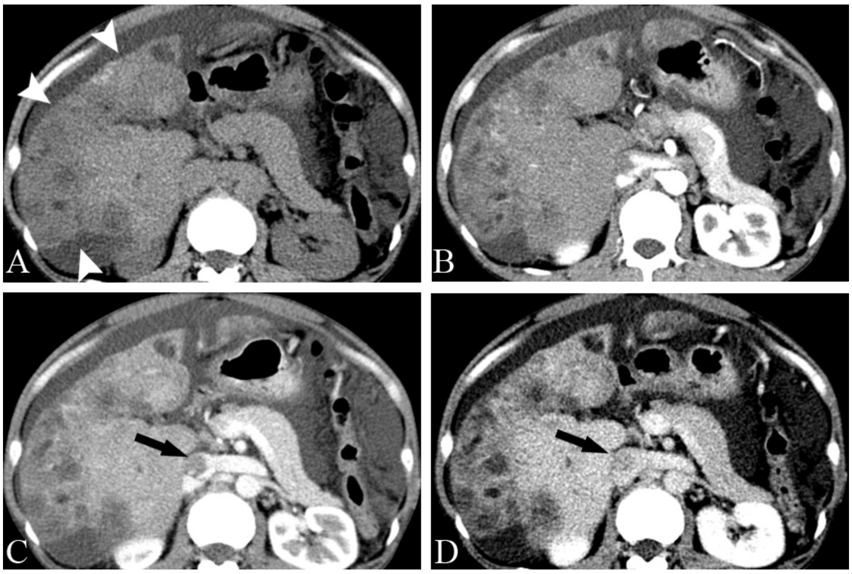

Figure S6. A 38-year-old man**.** (**A**) Axial unenhanced CT discovers multifocal hypodense lesions located under the hepatic capsule, with hepatic capsular retraction (*arrowheads*). (**B-D**) On axial contrast-enhanced CT, tumours showed mild enhancement on the axial arterial phase and centripetal progressive filling in the next two phases. The portal venous (**C**) and equilibrium phases (**D**) display tumour thrombi in the inferior vena cava (*arrows*).

Figure S7

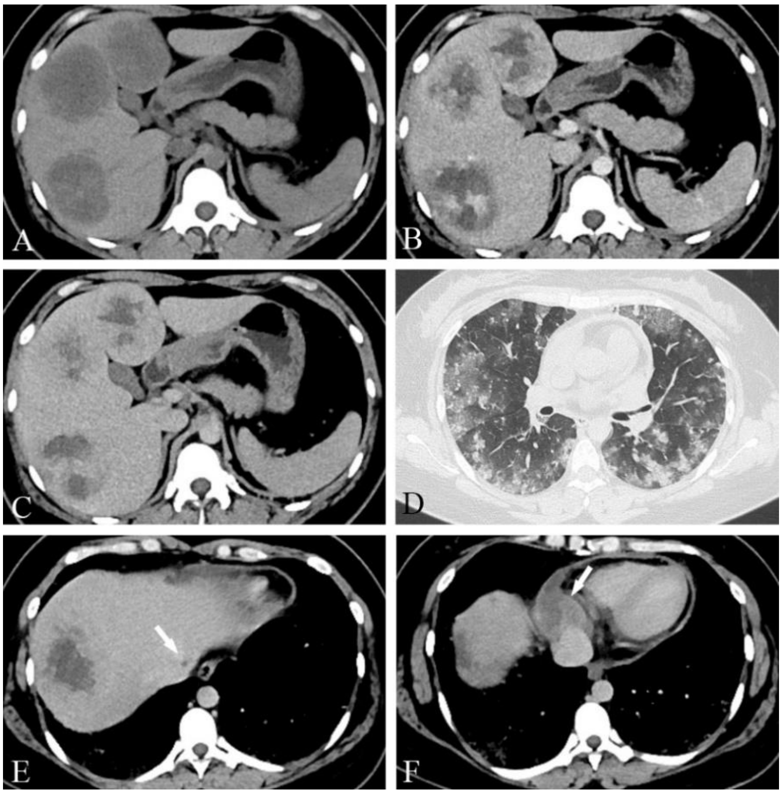

Figure S7. A 25-year-old woman. (**A**) Axial unenhanced CT discovers multifocal hypodense lesions in the liver. Tumours showed nodular peripheral enhancement with centripetal progressive filling on the portal venous and equilibrium phases (**B-C**). (**D-F)** On other chest and abdominal CT images, suspicious lesions were found in both lungs (**D**), superior vena cava (*arrow,* **E**), and right atrium (*arrow,* **F**).
